# Incomplete protection of the K_222_ polymorphism of the prion protein gene after intracerebral exposure to caprine BSE in goats: preliminary follow-up in S_127_ carriers

**DOI:** 10.1186/s13567-026-01760-8

**Published:** 2026-04-30

**Authors:** Diego Sola, José-Luis Pitarch, Belén Marín, Helen-Caroline Raksa, Alicia Otero, Francis Barillet, Frederic Bouvier, Rosa Bolea, Alex Bossers, Olivier Andreoletti, Jan Langeveld, Juan-José Badiola, Cristina Acín

**Affiliations:** 1 Centro de Investigación en Encefalopatías y Enfermedades Transmisibles Emergentes, Facultad de Veterinaria, IA2, Universidad de Zaragoza, C/Miguel Servet, 177, 50013 Saragossa, Spain; 2 UMR INRA-ENVT 1225, Interactions Hôte Agent Pathogène, Ecole Nationale Vétérinaire de Toulouse, Toulouse, France; 3 UE332 La Sapinière, INRAE, Osmoy, France; 4 Wageningen Bioveterinary Research, Wageningen University and Research, Lelystad, The Netherlands

**Keywords:** *PRNP*, BSE, goats, K_222_, S_127_

## Abstract

Among small ruminants, natural bovine spongiform encephalopathy (BSE) has been confirmed only in goats, raising concern about the possible entry of caprine BSE into the food chain. Genetic selection for *PRNP* alleles associated with resistance to classical scrapie, particularly K_222_ and S_127_, has been proposed as a control strategy, but their effect on caprine BSE is unclear. Nine Alpine goats carrying different *PRNP* genotypes at codons _222_ (Q/Q, Q/K, K/K) and _127_ (G/G, G/S) were intracerebrally inoculated with a second-passage caprine BSE isolate and monitored until terminal disease or censoring. Brain and selected peripheral tissues were examined for PrP^Sc^ by immunohistochemistry, western blotting, and conformation-dependent immunoassay, and lesion severity and PrP^Sc^ distribution were compared among genotypes. All goats lacking the S_127_ allele developed clinical disease with widespread PrP^Sc^ accumulation in the central nervous system. Compared with Q/Q_222_ goats, Q/K_222_ and K/K_222_ goats showed longer incubation periods and lower overall PrP^Sc^ and vacuolation scores, although disease still occurred in both genotypes after intracerebral challenge. Two G/S_127_ goats, one Q/Q_222_ and one Q/K_222_, remained clinically healthy and biopsy-negative at > 1400 days post-inoculation, but were right-censored because they were alive at last follow-up and had not undergone post mortem examination. PrP^Sc^ was also variably detected in lymphoreticular tissues and in the enteric and peripheral nervous systems, irrespective of genotype. Under these stringent challenge conditions, K_222_ delays disease onset but does not confer complete resistance. The prolonged survival of S_127_ carriers remains a preliminary, censored observation requiring post mortem confirmation.

## Introduction

Transmissible spongiform encephalopathies (TSE) are a group of fatal, neurodegenerative disorders caused by the accumulation in the central nervous system (CNS) of an abnormal isoform of a cellular protein (PrP^C^) called a prion (PrP^Sc^), causing progressive spongiform degeneration [1]. TSE affect several mammalian species, leading to similar diseases, the most significant being those affecting domestic ruminants: scrapie and bovine spongiform encephalopathy (BSE). Scrapie is a widespread non-zoonotic disease that commonly and naturally affects sheep and goats, while BSE in cattle is the most relevant for human health because of its zoonotic nature.

BSE represented one of the most important food crises of the last decades in Europe. It started in the UK, owing to consumption by cattle of feedstuff contaminated with prions [2–4]. The subsequent ingestion of meat products from those cattle caused variant Creutzfeldt–Jakob disease (vCJD) in humans [5–7]. EU efforts against BSE managed to almost eradicate the disease in the bovine population. However, the possibility that sheep and goats were also exposed to the same feedstuffs led to several experimental studies, whereby both species were successfully infected by intracerebral and oral routes [8–11], and even naturally in an experimental flock [12]. In fact, in 2005, the first case of truly natural BSE in a small ruminant was confirmed: a goat from France [13], and 1 year later, a retrospective study found a probable case in another goat from the UK [14] that was finally confirmed [15]. On the other hand, a recent study pointed out that small-ruminant BSE seems to propagate more efficiently than cattle BSE in human PrP murine models [16]. All these findings imply that BSE in small ruminants, especially goats, represents a high danger for human health, so different strategies must be considered to avoid possible infections.

It is well known that the development of a TSE depends largely on alterations in the *PRNP* gene of the host, which is responsible for encoding the cellular protein PrP^C^ [17]. The different polymorphisms may influence the conversion of PrP^C^ in PrP^Sc^, causing the animal to be more susceptible or resistant to the disease [18]. With this in mind, a comprehensive study of the ovine *PRNP* gene was performed, leading to the creation of a genetic breeding program to progressively increase the number of animals resistant to scrapie [19].

There is no active breeding program for the caprine population, and knowledge on the role of polymorphisms in TSE susceptibility is still very limited. Study of the caprine *PRNP* gene has revealed more than 16 silent mutations, and 42 amino acid substitutions [20–24]. In the case of BSE, few studies have addressed the genetic resistance in goats, with the polymorphism I142M being the only one associated with a slight increase of the incubation period after experimental BSE challenge [10]. Several studies of natural and experimental scrapie infection in goats showed how some allelic variations in the *PRNP* gene could modulate the susceptibility to or resistance against the disease. The presence of methionine (M) at codon 142 implies longer incubation periods, not only in experimental challenge [10] but also in natural outbreaks [22, 25, 26]. Some studies in naturally infected goats from Greece and France [25, 27, 28] have suggested that animals carrying arginine (R) at codon 143, histidine (H) at codon 154, or glutamine (Q) at codon 211 seem to be more resistant to the disease. One must bear in mind that the presence of histidine at codon 154 increases susceptibility to atypical scrapie [29]. Besides, two different polymorphisms at codon 146, found in Cypriot goats, conferred resistance to scrapie [23–30]. The presence of lysine (K) at codon _222_ had been associated with a protective effect in naturally infected goats from Italy and France [25–31].

A few years ago, an experiment was performed, inoculating goats carrying different genotypes (I/M_142_, R/H_154_, R/Q_211_, and Q/K_222_) with a goat natural scrapie isolate, by intracerebral and oral route [32]. The results confirmed that, although R/H_154_ and R/Q_211_ confer resistance against scrapie infection by oral route, only Q/K_222_ also appears to provide resistance to intracerebral infection. Together with this study, transgenic mice expressing the Q_222_ or K_222_ variant of goat PrP^C^ were challenged intracerebrally with different TSE isolates [33], showing that those mice expressing allele K_222_ were resistant to all goat scrapie and bovine BSE isolates but not to a caprine BSE isolate. In contrast with these results, experimental oral transmission with goat BSE [34] revealed a protective effect of the K_222_ allele in the oral susceptibility in goats. This made the Q222K polymorphism one of the main candidates for a breeding programme to eradicate TSEs in goats.

In the case of the G_127_S mutation, a study in UK goats suggested an association of the presence of serine (S) at codon _127_ with a decreased probability to develop clinical scrapie, prolonging the incubation period, but without modulation of the susceptibility to infection [22]. This was an associative study between *PRNP* genetics and susceptibility to scrapie in naturally infected scrapie goat herds, without experimental challenge with scrapie prions. Another study was also carried out by intracerebrally inoculating classical scrapie to G/G_127_ and G/S_127_ goats, observing a significant increase in incubation time of G/S_127_ animals (647–1333 dpi) but concluding that allele S_127_ is not protective against the disease [35].

The aim of this work was to evaluate how *PRNP* variants K_222_ and S_127_ modulate the outcome after intracerebral caprine BSE challenge in goats, focusing on survival/incubation, CNS pathology, and PrP^Sc^ distribution. We also report interim observations for two S_127_ carriers still under follow-up, for which post mortem confirmation is pending. Therefore, it is of great interest to characterize the caprine BSE agent, revealing its immunohistochemical features and molecular profiles, performing the techniques of immunohistochemistry and western blotting with several antibodies with different epitope recognition sites, as described in previous studies performed in sheep and goats affected by several TSE [14–36].

## Materials and methods

### Ethics statement

This study was performed in accordance with the Spanish Animal Protection Policy contained in the RD 53/2013, which complies with EU directive 2010/63/UE on the protection of animals used for experimental and other scientific purposes. The experimental protocol was approved by the Committee on the Ethics of Animal Experimentation of the University of Zaragoza (permit PI12/11).

### Animals

Eleven Alpine breed goats (10 males and 1 female) were enrolled and grouped according to *PRNP* genotype at codon _222_: one group consisting of six animals homozygous for glutamine Q/Q_222_, a second group of two animals heterozygous glutamine/lysine Q/K_222_, and a third group of three animals homozygous for lysine K/K_222_. Two goats (one Q/Q_222_ and one Q/K_222_) carried the G_127_S substitution and were heterozygous G/S_127_ (Table 1). Group sizes were constrained by the availability of rare genotypes, particularly K/K_222_ and G/S_127_. Nine goats (five Q/Q_222_, two Q/K_222_, and two K/K_222_) were intracerebrally inoculated; the remaining two goats (one Q/Q_222_ and one K/K_222_) served as non-inoculated negative controls and were followed in parallel. All male goats were sterilized except four: two Q/Q_222_ and two K/K_222_, which were kept intact for management reasons related to pairing with the female. Animals were kept throughout the duration of the experiment in facilities with biosafety level P3 at the Research Center in Encephalopathies and Emerging Infectious Diseases (University of Zaragoza), as required in case of experiments with BSE.

**Table 1:** **Incubation period and survival time of goats inoculated intracerebrally with caprine BSE**

Animal ID	Sex	Genotype		Incubation period (days)	Survival time (days)	Duration of clinical signs (days)
		Codon _127_	Codon _222_			
C1	Male	G/G	Q/Q	/	481*	/
C2	Male	G/G	Q/Q	470	501	31
C3	Male	G/G	Q/Q	470	506	36
C4	Male	G/G	Q/Q	470	513	43
C5	Male	G/S	Q/Q	/	/	/
C6	Male	G/G	Q/K	544	638	94
C7	Male	G/S	Q/K	/	/	/
C8	Female	G/G	K/K	679	716	37
C9	Male	G/G	K/K	709	733	24

ID, identification; G, glycine; Q, glutamine; S, serine; K, lysine. *Animal euthanized in absence of clear clinical signs owing to paralysis of hind limb

### Extraction and purification of genomic DNA

Genomic DNA was extracted from blood using a commercial QIAGEN^®^ kit (QIAamp DNA Mini Kit), following the manufacturer’s protocol. First, digestion of 10 min at 56 °C with proteinase K (20 ng/mL) lysed the cells of the tissue. Then, by using a membrane column, ethanol was added to assist the precipitation of DNA and its binding to the membrane. Finally, a series of washes was applied to remove debris and recover the purified genomic DNA.

### *PRNP* gene amplification and sequencing

The open reading frame (ORF) of the *PRNP* gene (750 bp) of all the animals in the study was amplified by performing a polymerase chain reaction (PCR) using the reagents of the commercial QIAGEN^®^ kit (HotStarTaq^®^ Master Mix Kit) and primers PrP8 (fwd) (5′-CAGGTTAACGATGGTGAAAAGCCACATAGG-3′) and PrP9 (rev) (5′-GGAATTCTATCCTACTATGAGAAAAATGAGG-3′) [37]. PCR reactions were purified using the vacuum manifold from Millipore^®^. Bidirectional sequencing was performed using the same PCR primers. Chromatograms were analyzed using BioEdit version 4.8.6.

### Intracerebral challenge (IC)

Inoculum was prepared with second-passage goat BSE brain tissue obtained from a wild-type *PRNP* goat (WT) (G/G_127_; Q/Q_222_). This goat had been intracerebrally (IC) inoculated with first-passage goat BSE derived from another wild-type goat, which had previously been intracerebrally inoculated with bovine BSE originating from a Dutch cow. Brain tissue (1 g) was homogenized with 10 mL normal sterile saline solution to final concentration of 10%. Nine animals were inoculated: five homozygous Q/Q_222_ goats, the two heterozygous Q/K_222_ animals, and two of the three homozygous K/K_222_ individuals; the remaining two goats were maintained as non-inoculated negative controls. Animals were anesthetized with ketamine (2–5 mg/kg IV) and diazepam (0.1–0.25 mg/kg IV), then an incision was made in the skin located 1 cm lateral to the midline between the frontal bones, equidistant from the lateral corner of the eye and the base of the cranial border of the ear. After trepanation, 0.5 mL inoculum was delivered intracerebrally using a hypodermic needle. Following the procedure, buprenorphine was administered as analgesic (0.005–0.01 mg/kg IM) every 8 h. Animals were monitored closely during recovery and received veterinary care as required.

### Clinical evaluation

Daily observation of animals was carried out, with a comprehensive neurological test performed once a month, taking photographs and recording video of all the animals. This test was described in a previous study about monitoring clinical signs in goats affected by TSE [38] and assessed posture, head carriage, abnormal movements (tremors, fasciculation), mental status, behavior, menace response, scratch/nibble test, body condition, gait, proprioception, reflexes, skin lesions, and hair loss.

### Tissue sample collection

Periodically, every 3 months, 30 mL blood was collected from all the animals using three different tubes of 10 mL: one with ethylenediaminetetraacetic acid (EDTA) anticoagulant to perform white cell extraction protocol, one with citrate dextrose anticoagulant for storing whole blood and obtaining plasma, and one without any reactant to obtain serum. All this material was stored for use in future research. Besides, recto-anal mucosa-associated lymphoid tissue (RAMALT) biopsies were obtained to assess PrP^Sc^ deposition. In addition, third-eyelid biopsies were collected as an ancillary lymphoid sampling site. Biopsies were processed and examined using the same immunohistochemical protocol as described below; samples were considered positive when specific PrP^Sc^ immunolabeling was observed within lymphoid follicles, and negative when no specific labeling was detected.

Animals were left to live to the terminal stage of the disease, at which time they were culled for reasons of animal welfare. All animals were euthanized with an intravenous overdose of pentobarbital sodium (Dolethal^®^). Once euthanized, a systematic and complete necropsy was performed, storing duplicate samples of all tissues collected: frozen at −80 °C and preserved in formaldehyde at 10%. The samples collected were: nervous tissue (cervical, thoracic and lumbar spinal cord; obex; pons; midbrain; cerebellum; frontal, parietal and occipital cortex; striatum; thalamus; hippocampus; trigeminal ganglia; optic chiasm; eye; nasal mucosa; and brachial and sciatic nerves); lymphoreticular tissue (third eyelid; tonsils; lymph nodes [retropharyngeal, mediastinal, mesenteric, iliac, pre-scapular, submandibular, popliteal and mammary]; ileal and jejunal Peyer patches; ileocaecal valve and spleen); digestive tissue (tongue, esophagus, rumen, reticulum, omasum, abomasum, duodenum, jejunum, ileum, cecum, rectum, liver and pancreas); and other tissues (lung, heart, skin, kidney, urinary bladder, adrenal gland, triceps brachii muscle, semitendinosus muscle, uterus, ovaries, mammary gland, and testicle).

### PrP^Sc^ immunohistochemical detection and hematoxylin–eosin stain

Sections (5 µm) of formaldehyde-fixed and paraffin-wax-embedded tissue samples were subjected to immunohistochemical diagnosis for TSE using two different antibodies: the mouse monoclonal antibody L42 (1:500; R-Biopharm, Darmstadt, Germany), which recognizes a region of the C terminus of the protein (_144_FGNDYEDRYYRENMYRYPNQVYY_166_) [39]; and mouse monoclonal antibody P4 (1:160; R-Biopharm, Darmstadt, Germany) [40], which recognizes a segment of the N-terminal domain of the flexible tail of PrP (_93_WGQGGSH_99_). The technical description has been described elsewhere [41]. In addition, only CNS tissue sections were stained with hematoxylin and eosin (HE), to observe histopathological lesions, with particular emphasis on vacuolation, which was scored subjectively from 0 (absent) to 3 (severe).

### PrP^Sc^ profile

The magnitude of accumulation of different PrP^Sc^ aggregates types, described in detail elsewhere [42], was scored subjectively from 0 (absent) to 3 (severe), in 24 specific areas of the brain and 3 of each spinal cord segment. The PrP^sc^ types considered were intraneuronal, intramicroglial, intrastrocytic, stellate, subpial, subependymal, perivascular, perivacuolar, particulate/coalescing, perineuronal, and linear. The areas of neuroparenchyma studied were the frontal cerebral cortex (grey and white matter), the corpus striatum (internal and external capsules; caudate, lenticular and accumbens nuclei; and septal area), the diencephalon (dorsal and ventral thalamus and hypothalamus), the midbrain (occulomotor and red nuclei, lateral geniculate body and substantia nigra), the cerebellum (grey and white matter), the pons (deep cerebellar, vestibular and facial nuclei), and the medulla at the obex (dorsal motor of the vagus [DMNV], hypoglossal, lateral cuneate, spinal tract of the trigeminal nerve and olivary nuclei, and the spinocerebellar tract). The average values for each PrP^Sc^ type from the different areas examined were used to construct the PrP^Sc^ profile, and the total PrP^Sc^ score for each sheep was calculated as the sum of these average values [42].

### Immunoblot analysis

Tissue samples stored at −80 °C were analyzed by western blot technique to determine the presence of PrP^res^ and to study the glycosylation pattern, using the Prionics^®^-Check WESTERN BSE commercial kit and following the manufacturer’s protocol. PrP^Sc^ immunodetection was performed with two antibodies: the mouse monoclonal antibody L42 (1:3000; R-Biopharm, Darmstadt, Germany), which recognizes a C-terminal region of the protein (144FGNDYEDRYYRENMYRYPNQVYY166) [39]; and mouse monoclonal antibody P4 (R-Biopharm, Darmstadt, Germany) [40], which recognizes a segment of the N-terminal domain of the flexible tail of PrP (_93_WGQGGSH_99_).

### PrP^Sc^ enzyme-linked immunosorbent assay (ELISA) detection

ELISA was performed in all samples collected by using the IDEXX HerdChek BSE-Scrapie Antigen EIA (IDEXX Laboratories, Westbrook, USA), according to the manufacturer’s instructions, and using the conjugate for cattle in each sample. Negative cutoff values were 0.165 absorbance units for the bovine conjugate.

## Results

### Incubation period and clinical stage

Unless otherwise stated, values are presented as mean ± standard deviation (SD).

Among Q/Q_222_ goats, three animals developed clinical signs at 470 days post inoculation (dpi) and were euthanized at 507 ± 6 dpi (Table 1). A fourth Q/Q_222_ goat (C1) was euthanized at 481 dpi because of hind limb paralysis without clear TSE-compatible clinical signs. The remaining Q/Q_222_ goat carrying G/S_127_ remained alive and clinically normal at the time of manuscript preparation (>1400 dpi) and was therefore censored. Among Q/K_222_ goats, one animal developed clinical signs at 544 dpi and was euthanized 94 days later (survival time 638 dpi), representing a 74-day prolongation of incubation relative to the Q/Q_222_ group. The second Q/K_222_ goat carrying G/S_127_ remained alive and clinically normal at last follow-up (>1400 dpi) and was therefore censored. Both inoculated K/K_222_ goats developed clinical signs at 694 ± 21 dpi and were euthanized 1 month later at 725 ± 12 dpi. Relative to Q/Q_222_ goats, the K/K_222_ genotype was associated with a 224 ± 21 day increase in incubation time; compared with the single clinically affected Q/K_222_ goat, the increase was 150 ± 21 days. Of the two non-inoculated controls, the K/K_222_ control was euthanized owing to lymphadenitis involving internal lymph nodes, whereas the Q/Q_222_ control remained alive; neither control showed neurological signs suggestive of TSE. Both animals did not present any clinical signs.

The periodic clinical examinations revealed that animals from the three groups developed similar clinical signs such as weakness, weight loss, increasingly aversive behavior, and depressed mental status. All clinically affected animals also developed a wide-based stance with asymmetrical ataxia, proprioceptive deficits, head tilt, head tremors, and a characteristic, forward and down position of the ears. Besides, an absent menace response was observed in two goats, one Q/Q_222_ and one K/K_222_. The evolution of the disease was very fast, and the animals were euthanized approximately 1 month later in all cases except in the animal Q/K_222_, which was euthanized approximately 3 months later. On the other hand, all periodic rectal biopsies were negative for PrP^sc^.

### PrS^sc^ phenotype and vacuolar profiling of caprine BSE in goats

**Figure 1:**
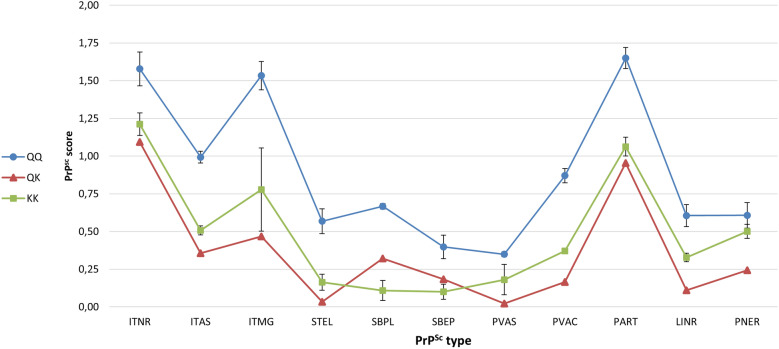
**Effect of *PRNP* genotype at codon**_**222**_ **on the PrP**^**Sc**^** profiles of goats inoculated intracerebrally with caprine BSE**. ITNR, intraneuronal; ITAS, intra-astrocytic; ITMG, intramicroglial; STEL, stellate; SBPL, subpial; SBEP, subependymal; PVAS, perivascular; PVAC, perivacuolar; PART, particulate/coalescing; LINR, linear; PNER, perineuronal. Error bars represent the standard error of the mean (SEM).

**Figure 2:**
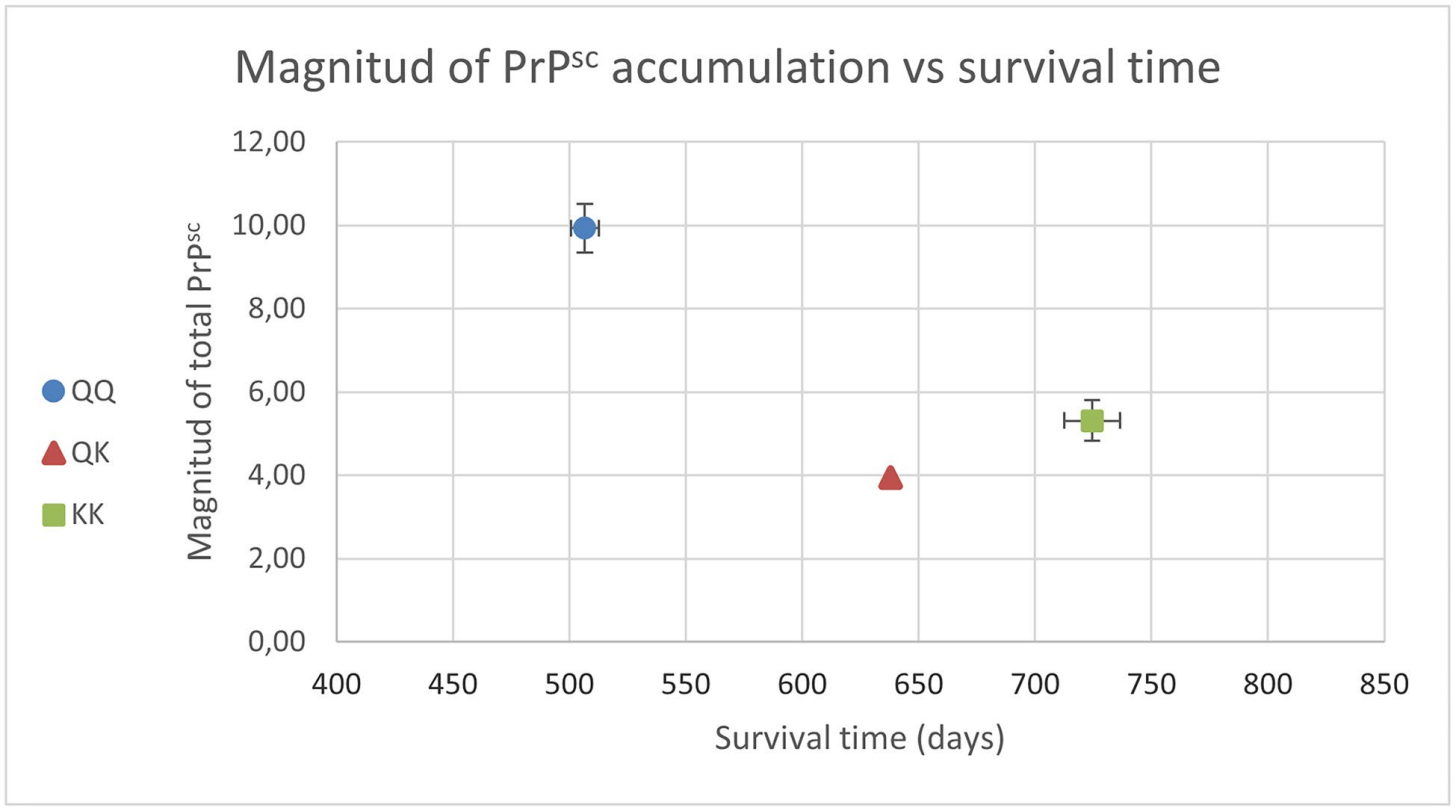
**Magnitude of PrP**^**Sc**^** accumulation and survival time in goats grouped by *PRNP* idem at codon **_**222**_ **after genotype.**

The phenotype of PrP^Sc^ accumulation in the neuroparenchyma of CNS was very similar in all three groups of animals, only showing differences concerning the magnitude of deposition, which varied depending on the genotype (Figure 1). The PrP^Sc^ profile was characterized by conspicuous intracellular PrP^Sc^ aggregates, mainly in neurons, but also in glial cells such as microglia and astrocytes. In case of extracellular deposits, the most prominent was particulate/coalescing, followed by some of the glia-associated types: stellate, subpial, perivacuolar, perineuronal, and linear. Other aggregates such as subependymal and perivascular were inconsistent. Q/Q_222_ animals showed high levels of PrP^Sc^ accumulation in all areas of the brain, mainly in corpus striatum, midbrain, and thalamus/hypothalamus, and to a lesser extend in cerebral cortex, cerebellum, and obex. The most affected nuclei were the lenticular and caudate nuclei in corpus striatum; substantia nigra and geniculate nucleus in midbrain; thalamus and hypothalamus; followed by dorsal motor nucleus of vagus nerve, spinal tract nucleus of the trigeminal nerve, and olivary nuclei in obex; cerebellum and cerebral grey matter. In case of animals carrying lysine at codon _222_, the neuroanatomical distribution of PrP^Sc^ aggregates was essentially the same, but with lower magnitude: 3.95 in case of Q/K_222_ goat and 5.31 ± 0.48 in K/K_222_ goats, compared with 9.82 ± 0.53 for Q/Q_222_ individuals (Figure 2).

As occurred with PrP^Sc^ deposition, the highest scores of vacuolation were registered in corpus striatum, midbrain, and thalamus/hypothalamus, affecting mainly lenticular and caudate nuclei in corpus striatum; substantia nigra and geniculate nucleus in midbrain; and thalamus.

### Peripheral distribution of PrS^sc^

**Figure 3:**
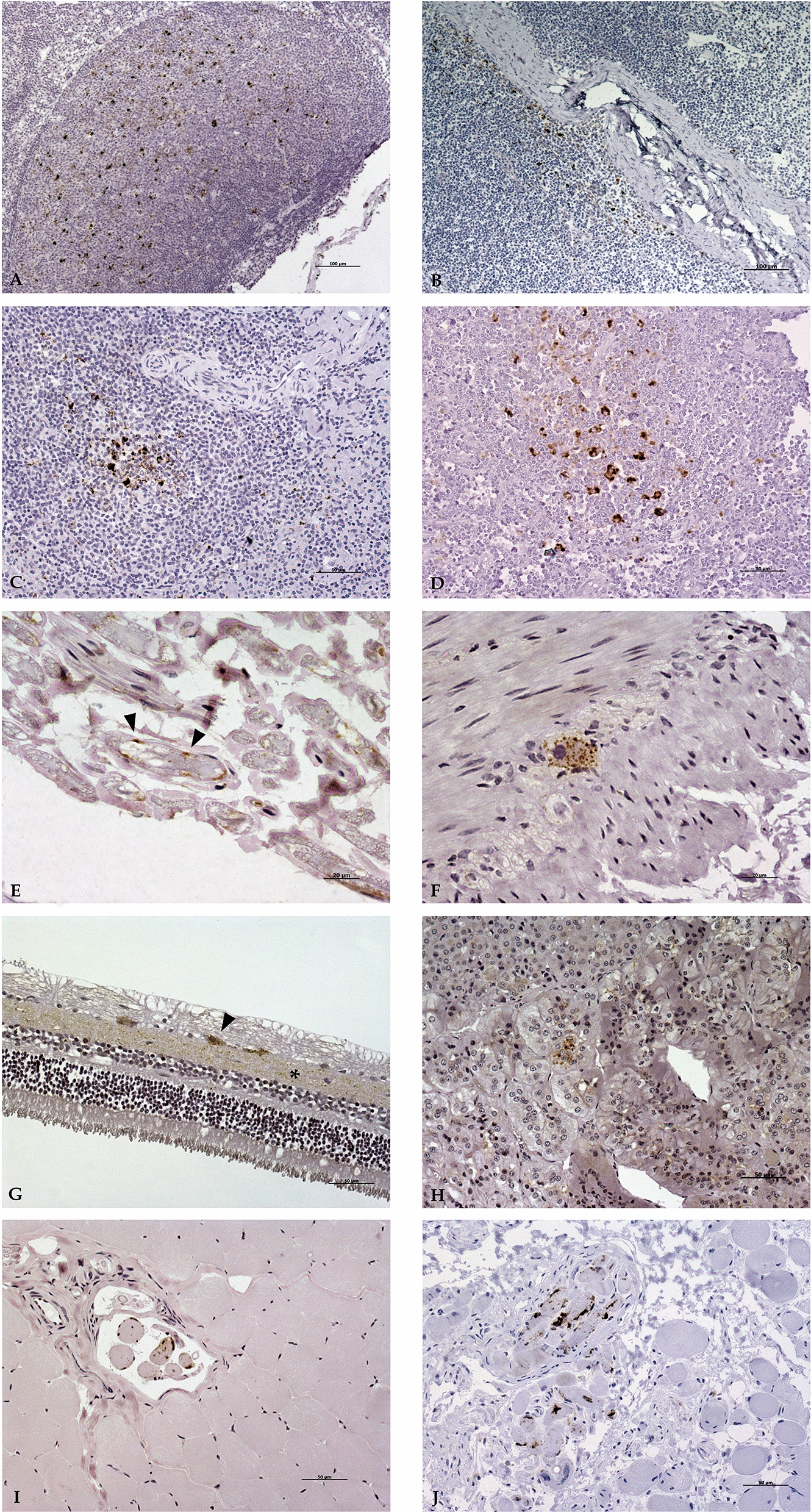
**Immunohistochemical detection of PrP**^**sc**^** aggregates in different locations using L42 antibody**. **A** Retropharyngeal lymph node of a Q/Q_222_ animal (10×). **B** Retropharyngeal lymph node of a K/K_222_ animal, with PrP^sc^ aggregates in the paracortical structures surrounding blood vessels (10×). **C** Spleen of the Q/K_222_ animal (20×). **D** Peyer patch in the ileocecal valve of a Q/Q_222_ animal (20×). **E** Brachial nerve of a K/K_222_ animal (40×). Periaxional PrP^sc^ aggregates can be observed (arrowheads). **F** Submucosal plexus at level of ileum of a Q/Q_222_ animal (40×). **G** Retina of a K/K_222_ animal (20×). PrP^sc^ immunolabeling is located in the inner plexiform layer (*) and inside the retinal ganglion cells (arrowhead). **H** Adrenal gland of a K/K_222_ animal (20×). Granular intracytoplasmic immunolabeling is associated with chromaffin cells. **I** Nerve ganglion located in the triceps brachii muscle of a K/K_222_ animal (20×). **J** Nerve fibers of the oculomotor muscles of a Q/Q_222_ animal (20×).

PrP^Sc^ deposition in the lymphoreticular system (Table 2) was observed in four animals in this study: two homozygous Q/Q_222_ animals, the heterozygous Q/K_222_ goat and one of the homozygous K/K_222_ animals. All of them presented marked PrP^Sc^ deposition in spleen and several lymph nodes, mainly in those located near the head (Figure 3A, C). However, no deposition was observed in the third eyelid in any of the animals. A single Q/Q_222_ goat presented PrP^Sc^ in the lymphoid follicles associated with the ileum and cecum mucosa (Figure 3D), and in a single follicle in the rectum. PrP^Sc^ accumulation mostly appeared in association with follicular dendritic cells (FDC) of light zone of the secondary follicles, but also in tingible body macrophages (TBM) in both light and dark zones of those follicles. In two animals, a homozygous Q/Q_222_ goat and a homozygous K/K_222_ animal, PrP^Sc^ aggregates were found outside of the follicles, in paracortical structures surrounding blood vessels (Figure 3B).

**Table 2:** **Distribution of PrP**^**sc**^** through central and peripheral nervous system and lymphoreticular system**

Central and peripheral nervous system								
Organ/tissue	Method	Q/Q_222_				Q/K_222_	K/K_222_	
		C1	C2	C3	C4	C6	C8	C9
Cerebral cortex	IHC	+	+	+	+	+	+	+
	ELISA	+	+	+	+	+	+	+
Corpus striatum	IHC	+	+	+	+	+	+	+
	ELISA	+	+	+	+	+	+	+
Midbrain	IHC	+	+	+	+	+	+	+
	ELISA	+	+	+	+	+	+	+
Cerebellum	IHC	+	+	+	+	+	+	+
	ELISA	+	+	+	+	+	+	+
Medulla oblongata	IHC	+	+	+	+	+	+	+
	ELISA	+	+	+	+	+	+	+
Cervical spinal cord	IHC	+	+	+	+	+	+	+
	ELISA	+	+	+	+	+	+	+
Thoracic spinal cord	IHC	+	+	+	+	+	+	+
	ELISA	+	+	+	+	+	+	+
Lumbar spinal cord	IHC	+	+	+	+	+	+	+
	ELISA	+	+	+	+	+	+	+
Eye	IHC	+	+	/	+	+	+	+
	ELISA	+	−	+	+	+	+	+
Brachial nerve	IHC	+	−	−	+	+	−	+
	ELISA	−	−	−	−	−	−	−
Sciatic nerve	IHC	−	−	−	/	−	/	−
	ELISA	−	−	−	−	−	−	−
Lymphoreticular system								
Organ/tissue	Method	Q/Q_222_				Q/K_222_	K/K_222_	
		C1	C2	C3	C4	C6	C8	C9
Third eyelid	IHC	−	/	−	−	−	−	−
	ELISA	−	−	−	−	−	−	−
Tonsil	IHC	−	+	−	+	+	−	−
	ELISA	−	−	−	+	−	−	−
Mandibular LN	IHC	−	+	−	+	+	+	−
	ELISA	−	+	−	−	−	+	−
Retropharingeal LN	IHC	−	+	−	+	+	+	−
	ELISA	−	+	−	−	−	+	−
Mesenteric LN	IHC	−	+	−	−	+	−	−
	ELISA	−	−	−	−	−	−	−
Spleen	IHC	−	+	+	+	+	+	−
	ELISA	−	+	−	+	+	+	−
Mediastinal LN	IHC	−	+	−	−	+	−	−
	ELISA	−	−	−	−	+	−	−
Prescapular LN	IHC	−	+	−	−	+	+	−
	ELISA	−	+	−	−	−	−	−
Iliac LN	IHC	−	+	−	−	+	−	−
	ELISA	−	−	−	−	+	−	−
Popliteal LN	IHC	−	+	−	−	−	−	−
	ELISA	−	+	−	−	−	+	−
Precrural LN	IHC	−	+	−	+	−	−	−
	ELISA	−	+	−	−	−	−	−

IHQ, immunohistochemistry; LN, lymph node

Regarding the peripheral nervous system (PNS) (Table 3), small PrP^Sc^ aggregates were observed in axons of the brachial nerve of four animals (Figure 3E), and in the sciatic nerve of a single goat, while conspicuous accumulation appeared in fibers of the enteric nervous system (ENS) lying near the smooth muscle of small and large intestines of three homozygous Q/Q_222_ goats (Figure 3F). PrP^Sc^ aggregates were found in nerve ganglions located in triceps brachii muscle of six animals (Figure 3I), and in semitendinosus muscle of a single K/K_222_ goat. Besides, abundant deposition appeared in nerve fibers of the oculomotor muscles of all animals (Figure 3J). Finally, two Q/Q_222_ goats and one of the K/K_222_ animals presented PrP^Sc^ deposition in the medulla of the adrenal gland (Figure 3H).

**Table 3:** **Distribution of PrP**^**sc**^** through gastrointestinal tract and other tissues**

Gastrointestinal tract								
Organ/tissue	Method	Q/Q_222_				Q/K_222_	K/K_222_	
		C1	C2	C3	C4	C6	C8	C9
Esophagus	IHC (L/N)	−/−	−/−	−/−	−/−	−/−	−/−	−/−
	ELISA	−	−	−	+	−	−	−
Rumen	IHC (L/N)	−/−	−/−	−/−	−/−	−/−	−/−	−/−
	ELISA	−	−	−	−	−	−	−
Omasum	IHC (L/N)	−/−	−/−	−/−	−/−	−/−	−/−	−/−
	ELISA	−	−	−	−	−	−	−
Reticulum	IHC (L/N)	−/−	−/−	−/−	−/−	−/−	−/−	−/−
	ELISA	−	−	−	−	−	−	−
Abomasum	IHC (L/N)	−/−	−/−	−/−	−/−	−/−	−/−	−/−
	ELISA	−	−	−	−	−	−	−
Duodenum	IHC (L/N)	−/−	−/+	−/+	−/−	−/−	−/−	−/−
	ELISA	−	−	−	−	−	−	−
Jejunum	IHC (L/N)	−/−	−/+	−/−	−/+	−/−	−/−	−/−
	ELISA	−	−	−	−	−	−	−
Ileum	IHC (L/N)	−/−	+/+	−/+	−/+	−/−	−/−	−/−
	ELISA	−	−	−	−	−	−	−
Ileocecal valve	IHC (L/N)	−/−	+/+	−/+	−/−	−/−	−/−	−/−
	ELISA	−	−	−	−	−	−	−
Cecum	IHC (L/N)	−/−	+/+	−/−	−/−	−/−	−/−	−/−
	ELISA	−	−	−	−	−	−	−
Colon	IHC (L/N)	−/−	−/+	−/+	−/+	−/−	−/−	−/−
	ELISA	−	−	−	−	−	−	−
Rectum	IHC (L/N)	−/−	±	−/+	−/−	−/−	−/−	−/−
	ELISA	−	−	−	−	−	−	−
Other tissues								
Organ/tissue	Method	Q/Q_222_				Q/K_222_	K/K_222_	
		C1	C2	C3	C4	C6	C8	C9
Extraocular muscles	IHC	+	+	+	+	+	+	+
	ELISA	−	−	−	−	−	−	−
Tongue	IHC	−	−	−	−	−	−	−
	ELISA	−	−	−	−	−	−	−
Heart	IHC	−	−	−	−	−	−	−
	ELISA	−	−	−	−	−	−	−
Triceps brachii muscle	IHC	+	−	+	+	+	+	+
	ELISA	−	−	−	−	−	−	−
Semitendinosus muscle	IHC	−	−	−	−	−	+	+
	ELISA	−	−	−	−	−	+	−
Kidney	IHC	−	−	−	−	−	−	−
	ELISA	−	−	−	−	−	−	−
Liver	IHC	−	−	−	−	−	−	−
	ELISA	−	−	−	−	−	−	−
Pancreas	IHC	−	−	−	−	−	−	−
	ELISA	−	−	−	−	−	−	−
Lung	IHC	−	−	−	−	−	−	−
	ELISA	−	−	−	−	−	−	−
Urinary bladder	IHC	−	−	−	−	−	−	−
	ELISA	−	−	−	−	−	−	−
Adrenal gland	IHC	−	+	+	−	−	−	+
	ELISA	−	−	+	−	−	−	+
Mammary gland	IHC	/	/	/	/	/	/	−
	ELISA	/	/	/	/	/	/	−
Skin	IHC	−	−	−	−	−	−	−
	ELISA	−	−	−	−	−	−	−

IHQ, immunohistochemistry; L, presence of PrP^Sc^ deposition associated to lymphoid tissue: N, presence of PrP^Sc^ deposition associated to nerve fibers of the ENS./, no sample

### Characterization of the agent of caprine BSE

**Figure 4:**
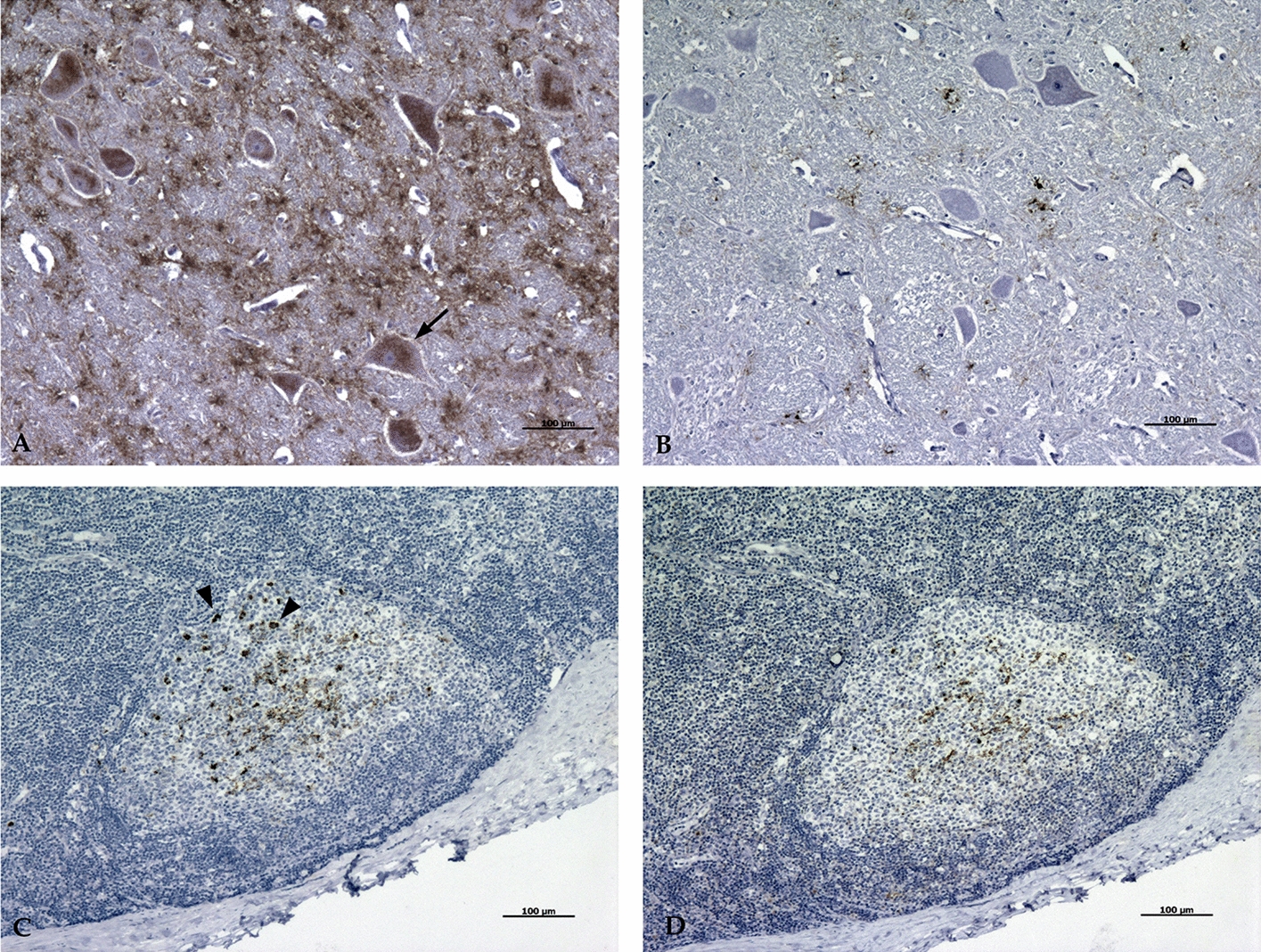
**Differentiation of labeling between antibody L42 and antibody P4**. **A** Antibody L42 labeled intraneuronal PrP^sc^ (Arrow) in the CNS (10×), **C** intra-TBM PrP^sc^ (arrowheads) in the LRS (10×), **B**–**D** Antibody P4 does not reveal both types of PrP^sc^ (10×). CNS, central nervous system; LRS, lymphoreticular system; TBM, tingible body macrophages.

Immunohistochemistry of CNS performed with C-terminal antibody L42 showed intraglial and intraneuronal labeling of PrP^Sc^ aggregates in all animals, in contrast to N-terminal antibody P4, which only showed light extracellular fine punctate or diffuse labeling (Figure 4A, C). In case of lymphoreticular system, L42 labeled both FDC- and TBM-associated PrP^Sc^, whereas with P4, TBM-associated immunolabeling is not apparent (Figure 4B, D).

**Figure 5:**
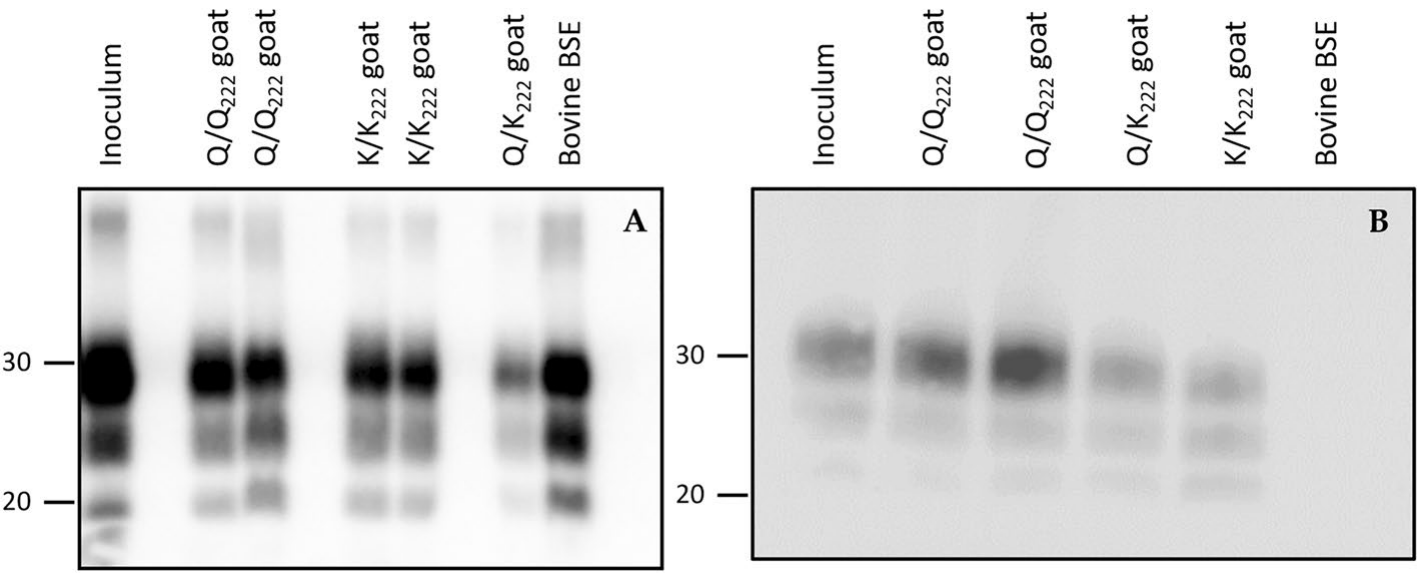
**PrP**^**sc**^** western blot patterns from intracerebrally inoculated goats with caprine BSE**. PrP^sc^ detected using L42 (**A**) and P4 (**B**). Lanes (left to right) (**A**): inoculum; Q/Q_222_ goat C2; Q/Q_222_ goat C3; K/K_222_ goat C8; K/K_222_ goat C9; Q/K_222_ goat C6; bovine BSE reference control. Molecular masses (kDa) are shown on the left. Lanes (left to right) (**B**): inoculum; Q/Q_222_ goat C2; Q/Q_222_ goat C3; Q/K_222_ goat C6; K/K_222_ goat C8; bovine BSE reference control. Molecular masses (kDa) are shown on the left.

WB technique revealed patterns of PrP^Sc^ of caprine BSE, using either L42 or P4 with some features overlapping published bovine BSE profiles in small ruminants, but also notable differences in P4 reactivity (Figure 5).

## Discussion

Recent experimental and field studies have consistently shown that the K_222_ allele is one of the most effective *PRNP* variants for reducing susceptibility to classical scrapie in goats and for delaying or preventing disease after oral exposure to caprine BSE [32–34]. In contrast, the G_127_S substitution has been associated with reduced incidence and prolonged incubation of classical scrapie in some populations, but it does not abolish infection and has therefore not been considered a truly protective allele [35]. Our study extends this work by specifically evaluating these two polymorphisms under the highly stringent conditions of intracerebral challenge with a caprine BSE isolate.

It should be noted that this study has important limitations that frame its interpretation: (i) small group sizes per genotype, (ii) intracerebral inoculation is a maximal, non-natural exposure route that bypasses peripheral barriers, and (iii) the two S_127_ carriers are still alive; consequently, their outcomes are right-censored, and the absence of post mortem examination means that subclinical infection and tissue PrP^Sc^ accumulation cannot be excluded. Therefore, any statements regarding S_127_ are preliminary and hypothesis-generating.

In agreement with previous data, the presence of lysine at codon _222_ clearly modulated the disease course in our experiment. All inoculated goats lacking the S_127_ allele eventually developed clinical caprine BSE, but Q/K_222_ and K/K_222_ animals showed longer incubation periods and lower global PrP^Sc^ and vacuolation scores than Q/Q_222_ goats. These observations support the view that K_222_ reduces susceptibility and slows within-host propagation of caprine BSE, yet does not provide absolute resistance under direct IC exposure, in either heterozygous or homozygous animals. This is consistent with earlier work in K_222_-transgenic mice, which reported resistance to cattle BSE isolates but not to caprine BSE [33]. Together with previous oral challenge experiments in goats [32–34], our data indicate that K_222_ is highly beneficial but that its protective effect is both strain- and route-dependent.

In goats challenged with classical scrapie, allele K_222_ almost completely prevented clinical disease in heterozygotes after oral inoculation, while only a minority of homozygous K/K_222_ animals developed disease and did so after very long incubation periods [32–34]. In a similar way, oral challenge with caprine BSE resulted in prolonged survival of Q/K_222_ goats and very low residual infectivity in brain and muscle at necropsy [34]. By contrast, in our IC caprine BSE experiment, differences in survival between genotypes were reduced: incubation periods in Q/K_222_ and K/K_222_ goats were only modestly longer than in Q/Q_222_ animals and remained markedly shorter than those reported for K/K_222_ goats infected orally with caprine BSE or intracerebrally with classical scrapie [32–34]. Since the IC route bypasses peripheral barriers and delivers a high dose directly into the brain, these findings probably reflect the limits of K_222_-mediated protection under maximal challenge rather than a lack of effect of the allele itself.

Two goats in our cohort carried the G_127_S substitution, one on a Q/Q_222_ and one on a Q/K_222_ background. Both animals remained clinically normal at the end of the observation period and showed no detectable PrP^Sc^ in serial rectal and third-eyelid biopsies, despite survival well beyond the average incubation time of the other genotypes. This pattern is consistent with delayed clinical onset in these two individuals; however, because both animals remain alive and have not undergone post mortem examination, subclinical infection cannot be excluded. This contrasts with data for classical scrapie, where the S_127_ allele did not prevent infection and was not considered protective [35], and underscores the need for continued follow-up and complete tissue analysis before recommending S_127_ for breeding programmes against BSE.

The PrP^Sc^ phenotype and lesion profiles that we observed in IC-challenged goats were broadly consistent across *PRNP* genotypes. All clinically affected animals showed abundant intracellular PrP^Sc^, predominantly intraneuronal and intraglial, with particulate, granular, and perineuronal deposits, and the highest scores in corpus striatum, midbrain, and thalamus. Goats carrying K_222_ had a similar neuroanatomical distribution to Q/Q_222_ animals but lower overall PrP^Sc^ scores, in line with their longer survival times. These findings parallel previous descriptions of IC bovine BSE and caprine BSE in small ruminants [43, 44] and support the idea that K_222_ alters the kinetics rather than the topography of PrP^Sc^ accumulation. This is because, although PrP^Sc^ accumulation is closely related to disease, clinical expression does not necessarily vary linearly with total PrP^Sc^ load as measured by IHC.

When compared with historical IC bovine BSE challenges in goats, which produced mean incubation periods of approximately 530–570 days in wild-type animals (Q/Q_222_) [9, 10], the caprine BSE isolate used here appears at least as aggressive. Nonetheless, we did not observe major qualitative differences in lesion targeting between caprine and bovine BSE in goats, suggesting that the strain adaptation primarily affects incubation time and biochemical properties rather than gross neuroanatomical tropism.

Outside the central nervous system, PrP^Sc^ was found in components of the lymphoreticular, enteric, and peripheral nervous systems, but its distribution was heterogeneous and did not show a clear relationship with *PRNP* genotype. Consistent with previous work in goats orally challenged with caprine BSE [45], lymph node involvement was most evident in head and upper-body nodes, with more limited and inconsistent detection in distal lymphoid tissues. Only one goat showed PrP^Sc^ in gut-associated lymphoid tissue of ileum, cecum, or rectum, and routine rectal and third-eyelid biopsies remained negative in all animals throughout the incubation period. Multiple studies in naturally infected goats with classical scrapie report that RAMALT/rectal biopsy has variable—and in many settings limited—diagnostic sensitivity, influenced by disease stage, follicle yield, strain/tissue tropism, and host factors [46, 47]. In line with this literature, our serial RAMALT and third-eyelid biopsies remained negative under the present experimental conditions, indicating that these lymphoid biopsies may have limited sensitivity for ante mortem detection of caprine BSE in this intracerebral challenge model. Importantly, negative biopsies should not be interpreted as evidence of absence of infection, and performance may differ under oral exposure or for other strains.

A notable finding was the consistent detection of PrP^Sc^ in peripheral nerves and small ganglia embedded within limb and extraocular muscles, including in goats with relatively low lymphoreticular involvement. Although we did not systematically quantify infectivity and only quantify PrP^Sc^ accumulation, this neuromuscular localization supports the view that skeletal muscle cannot be considered completely free of prion risk in caprine BSE and should be taken into account in future risk assessment exercises and discussions on specified risk materials.

Finally, combining immunohistochemistry and western blotting with antibodies recognizing different PrP epitopes provided further insight into the biological properties of the caprine BSE agent. As in previous descriptions of bovine BSE in sheep and goats, N-terminal antibody P4 showed limited labeling of intraneuronal and intraglial deposits by immunohistochemistry, a feature classically associated with bovine BSE-like strains [14, 48–50]. In contrast, western blot analysis with the same antibody yielded a strong signal with a molecular mass and glycoform profile closer to that of classical scrapie than to cattle BSE, whereas earlier work reported reduced P4 reactivity for bovine BSE in small ruminants [36, 40, 49]. These mixed biochemical and immunohistochemical characteristics, together with the incubation data, are consistent with a prion strain that has undergone partial adaptation to the caprine host and now occupies an intermediate position between classical scrapie and bovine BSE.

## Conclusions

Our work has certain limitations that we have already discussed above. However, taken together with previous field observations and experimental infections [32–35], our findings reinforce the central role of K_222_ as a key target for breeding programmes against classical scrapie and orally acquired BSE, while highlighting that this allele alone may not be sufficient to provide broad-spectrum protection against all caprine prion strains. The two S_127_ carriers are still being monitored; a final clinical evaluation and a complete analysis of post mortem tissues are required before conclusions can be drawn about S_127_, and therefore further studies are needed in the future.

## Data Availability

All data generated or analyzed during this study are included in this published article.
